# A Segment Anything Model-based tool for semi-automated behavioural analysis of *Drosophila* and other model organisms

**DOI:** 10.1242/dmm.052631

**Published:** 2026-02-24

**Authors:** Sarah Mele, Joshua Millward, Long Nguyen, Natasha Ruth, Jemma Gasperoni, Sebastian Dworkin, Zhen He, Travis K. Johnson

**Affiliations:** ^1^ Department of Biochemistry and Chemistry, and La Trobe Institute for Molecular Sciences, La Trobe University, Bundoora, VIC 3086, Australia; ^2^ School of Computing, Engineering and Mathematical Sciences, La Trobe University, Bundoora, VIC 3086, Australia; ^3^ Department of Microbiology, Anatomy, Physiology and Pharmacology, and La Trobe Institute for Molecular Sciences, La Trobe University, Bundoora, VIC 3086, Australia

**Keywords:** Segmentation, Tracking, SAM2 model, Behaviour, *Drosophila melanogaster*, Zebrafish, *Danio rerio*

## Abstract

Quantitative behavioural analysis is a powerful approach for linking genotype to phenotype, but many existing tools require specialised hardware, extensive preprocessing or coding expertise. We present Segment Anything Model for Behavioural Analysis (SAMBA), an open-access, Google Colab-based pipeline that harnesses the Segment Anything Model 2 (SAM2) for accurate, semi-automated tracking without thresholding or background subtraction. With minimal user input, SAMBA extracts movement parameters, detects behavioural states and supports batch processing. Validating SAMBA in three *Drosophila melanogaster* models of human neurological disease revealed impaired locomotion, reduced speed and altered decision-making. We further demonstrated adaptability to adult *Drosophila* and larval zebrafish, underscoring its cross-species utility. By combining foundation-model segmentation with an accessible interface, SAMBA lowers technical barriers to analysing motor pattern defects and is readily extendable to diverse model organisms, life stages and experimental paradigms. This flexibility positions SAMBA as a valuable platform for accelerating disease mechanism studies, genetic screens and preclinical testing.

## Teaser

**Summary:** We present an easy-to-use, open-access tool designed for *Drosophila* larval movement analysis and readily customised for other applications, enabling rapid assessment of organism behaviour without specialised equipment or coding expertise.

## INTRODUCTION

The fruit fly *Drosophila melanogaster* is a powerful genetic model organism that has contributed significantly to our understanding of neurobiology and diseases that impact the nervous system (Bellen et al., 2010; Jennings, 2011). A hallmark of many neurodegenerative disorders is progressive motor impairment, and the movement patterns of *Drosophila* larvae with equivalent genetic lesions reflect this. Larval locomotion normally follows predictable, stereotyped patterns, making deviations such as indecision or frequent changes in direction readily detectable (Jakubowski et al., 2012; Otto et al., 2018). Methods for examining larval locomotion patterns are particularly valuable in cases in which diseases induce early lethality and preclude adult analysis. Assessing larval locomotion ability is also relevant for drug and toxicology experiments to determine adverse effects on neurological function (Li et al., 2019).

Recent advances in deep learning have transformed image segmentation across diverse domains including medical imaging (Chan et al., 2020), autonomous driving (Gupta et al., 2021) and agriculture (Logeshwaran et al., 2024), by reducing the burden of existing manual processes. However, applying these traditional segmentation models typically requires large, annotated datasets trained for a specific task, which can make them time consuming and labour intensive to develop. Foundation models such as Meta's Segment Anything Model 2 (SAM2) overcome these constraints by being pre-trained on vast, heterogeneous datasets, enabling generalisation across domains without retraining (Ravi et al., 2024 preprint). In particular, SAM2 can segment objects with minimal prompting, even in complex visual environments, making it well suited to biological applications without extensive computer vision expertise.

Current approaches to larval tracking require binarised or thresholded inputs, or depend on specific imaging conditions or hardware setups (e.g. wrMTrck, FIMtrack). Although these tools have enabled detailed behavioural studies, there are limitations to their accessibility. Here, we present Segment Anything Model for Behavioural Analysis (SAMBA), an open-access, semi-automated pipeline that uses SAM2 to track and quantify movement. It functions directly on raw video files without the need for background subtraction. SAMBA is implemented in Google Colab, requiring no installation or coding skills, and supports batch processing. It extracts spatial and kinematic parameters, detects behavioural states such as pausing and head-casting, and outputs publication-ready summary data alongside raw data.

To illustrate its utility, we applied SAMBA to three *Drosophila* models of neurometabolic disorders: 3-hydroxyisobutyryl-CoA hydrolase (HIBCH) deficiency, maple syrup urine disease (MSUD) and isolated sulfite oxidase deficiency (ISOD) (Blackburn et al., 2017; Claerhout et al., 2017; Taura et al., 2023). We demonstrate that SAMBA captures both gross locomotor deficits and altered decision-making behaviours in mutant larvae, providing phenotypic resolution beyond distance and speed metrics. We also show its adaptability to adult *Drosophila* and larval zebrafish, underscoring cross-species potential for behavioural phenotyping in genetic, pharmacological and environmental studies. By combining the generalisation power of a foundation model with a user-friendly interface, SAMBA lowers technical barriers to detect motor pattern defects. Its flexibility makes it a valuable resource for researchers studying disease mechanisms, screening candidate therapies and characterising behavioural phenotypes across a broad range of model organisms.

## RESULTS

### Development of SAMBA for semi-automated larval tracking

**Fig. 1.**
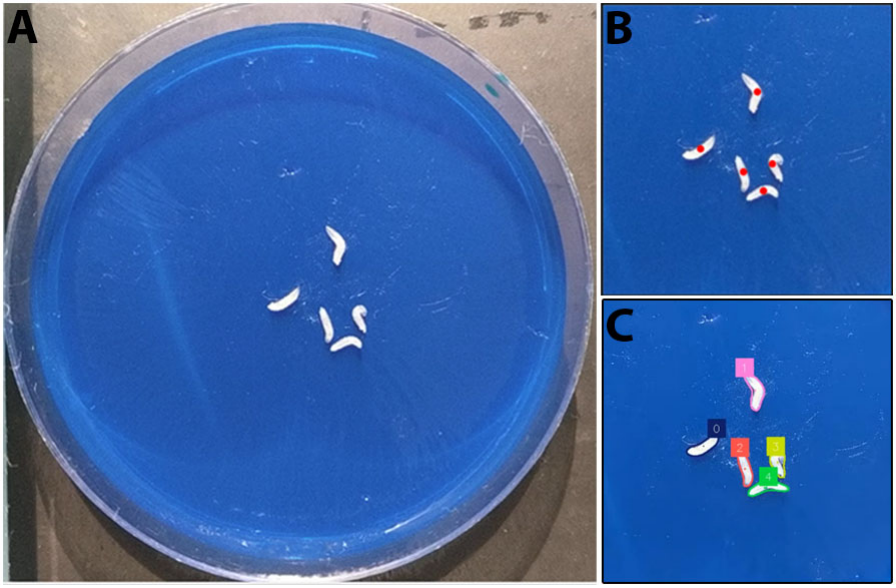
**Manual larva selection via point prompting.** (A) Frame 1 of a video of the larval arena used to apply Segment Anything Model 2 (SAM2) to larval locomotion. (B) User-provided point prompts (red dots) to identify objects of interest. (C) Output masks from SAM2 with a colour-coded ID added to each larva.

To create SAMBA, we adapted the pre-trained model SAM2 to analyse larval locomotion with minimal user input in the Google Colab environment. The notebook can be accessed via the GitHub repository (https://github.com/johnsonflygroup/SAMBA). To annotate an entire video, SAMBA requires a single frame to be annotated with point prompts for all objects of interest and is capable of forward- and backward-time tracking. This allows rapid, flexible segmentation of larvae across diverse imaging conditions. We based its development on a set of 100 3-min-long video recordings of five late third-instar wild-type *Drosophila* larvae crawling in a circular arena of 60 mm diameter (Fig. 1). However, the approach of manually selecting each object of interest and feeding it into SAM2 should generalise to any animal tracking application without the need for training a dedicated object-specific detector. The tool includes an interactive calibration step to convert pixel distances into millimetres, ensuring that outputs are directly interpretable in standard units provided that the camera is mounted perpendicular to the plane of object movement. From each segmented frame, SAMBA extracts position, orientation and shape data, which are compiled into raw and summary CSV files along with annotated videos and track images (Table 1). The raw CSV files contain per-frame *x*, *y* coordinates, ellipse major and minor axes, ellipse major/minor ratio and ellipse angle (degrees) for each object. Users requiring more advanced analysis could extract more specific behavioural metrics in other software packages using these measurements. We recommend running SAMBA on the Google Colab A100 or L4 GPU, which can process 3-min videos tracking five objects in ∼5 and 10 min, respectively [720p, ten frames per second (fps)]. In contrast, the freely available T4 GPU requires >1 h to process an equivalent video.

**Table 1.** Output files from SAMBA

Output folder	Output filenames	Description
Raw_frames	0.csv…n.csv	Raw data for each frame, including (*x*, *y*) coordinates, polygon size, ellipse major and minor axes, ellipse major/minor ratio, ellipse angle (degrees), major/minor ratio *z*-score, elongated/bent state, distance and speed for each object.
Problematic_frames	0.csv…n.csv*	Frames copied from raw_frames that contain aberrant movement behaviour for each object.
Good_frames	0.csv…n.csv	Frames copied from raw_frames that contain no problematic frames (aberrant movement behaviour) for each object.
Paths	0.csv…n.png	Movement tracings for each object. Includes highlighting for elongated and bent states.
−	output.csv	Aggregated data from good_frames.
−	tracking.mp4	An annotated version of the original uploaded video with colour-coded, labelled polygonal overlays on each object.
Problematic_frames_visualisation	0.jpg…n.jpg	Size and speed versus time scatterplots with overlay of removed problematic frames.

*May not be present for all tracked objects. SAMBA, Segment Anything Model for Behavioural Analysis.

**Fig. 2.**
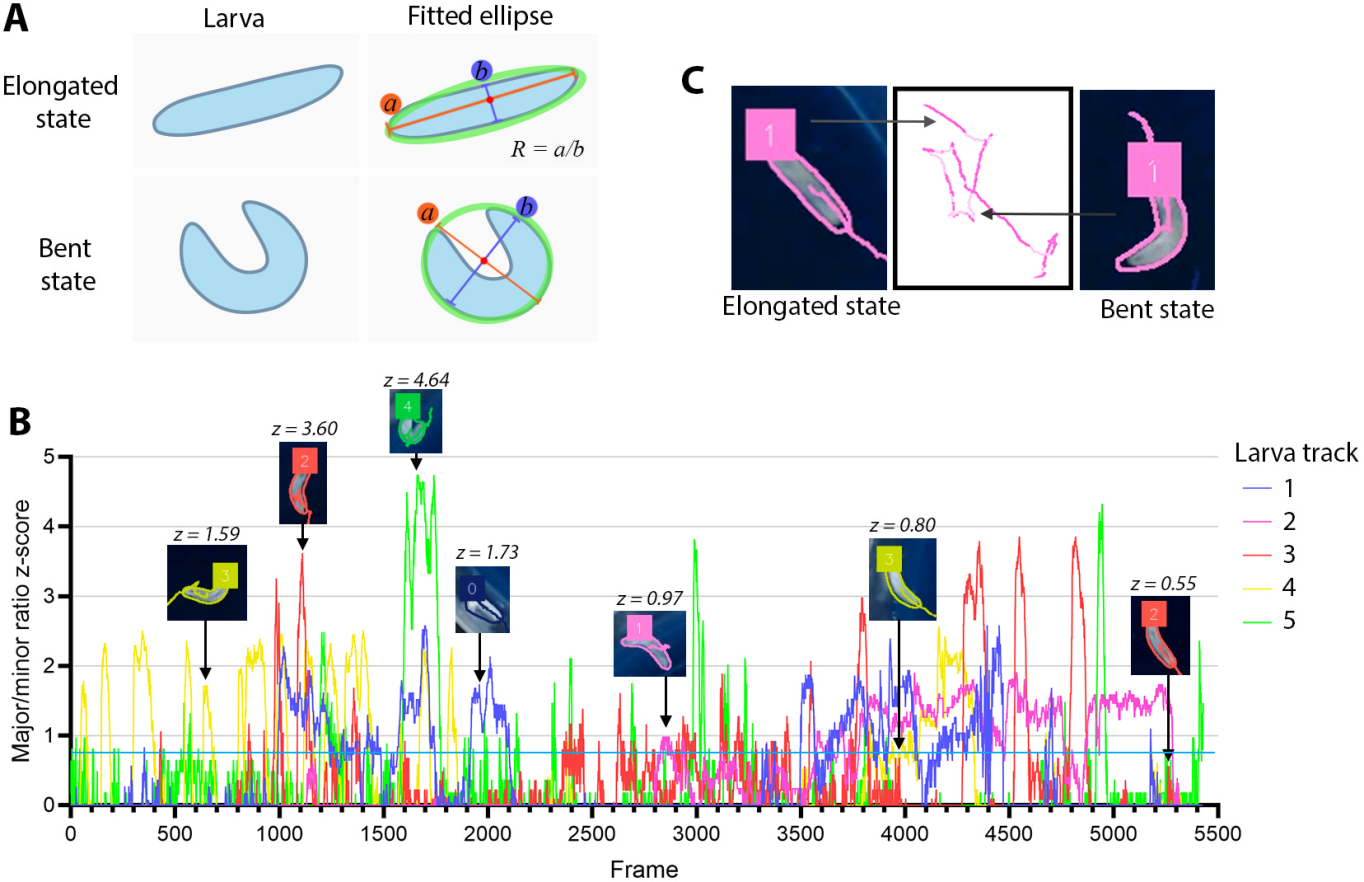
**Characterisation of larval shape.** (A) Schematic of how larvae are determined to be in a bent state using ellipses and the major-to-minor axis ratio (*R*=*a*/*b*, where ‘*a*’ represents the major axis and ‘*b*’ represents the minor axis). (B) Plotted *z*-scores of *R* for five larvae. Examples of larval orientations at different *z*-scores are overlaid. The cutoff for the ‘bent’ shape was visually determined to be *z*≥0.8. (C) Output images of larval tracks show darker-coloured tracks when a larva is elongated and a lighter colour when it is bent.

*Drosophila* larval behaviours include running, which is a period of forward movement or uninterrupted crawling; pausing, when the larva ceases forward motion; and head casting, where the larva sweeps its head side-to-side to sample its environment and make directional decisions (Berrigan and Pepin, 1995). Larvae also have foraging patterns dependent on strain, such as the traversal of a large area, a rover, or a small area, a sitter (Sokolowski, 1980). To quantify these behaviours, we sought to classify larval posture into ‘elongated’ or ‘bent’ states based on the ratio of ellipse major to minor axes (Fig. 2A). These posture states reflect the running versus head-casting behaviours, allowing interpretation of larval decision-making ability (Fig. 2B,C).

### Data quality control

**Fig. 3.**
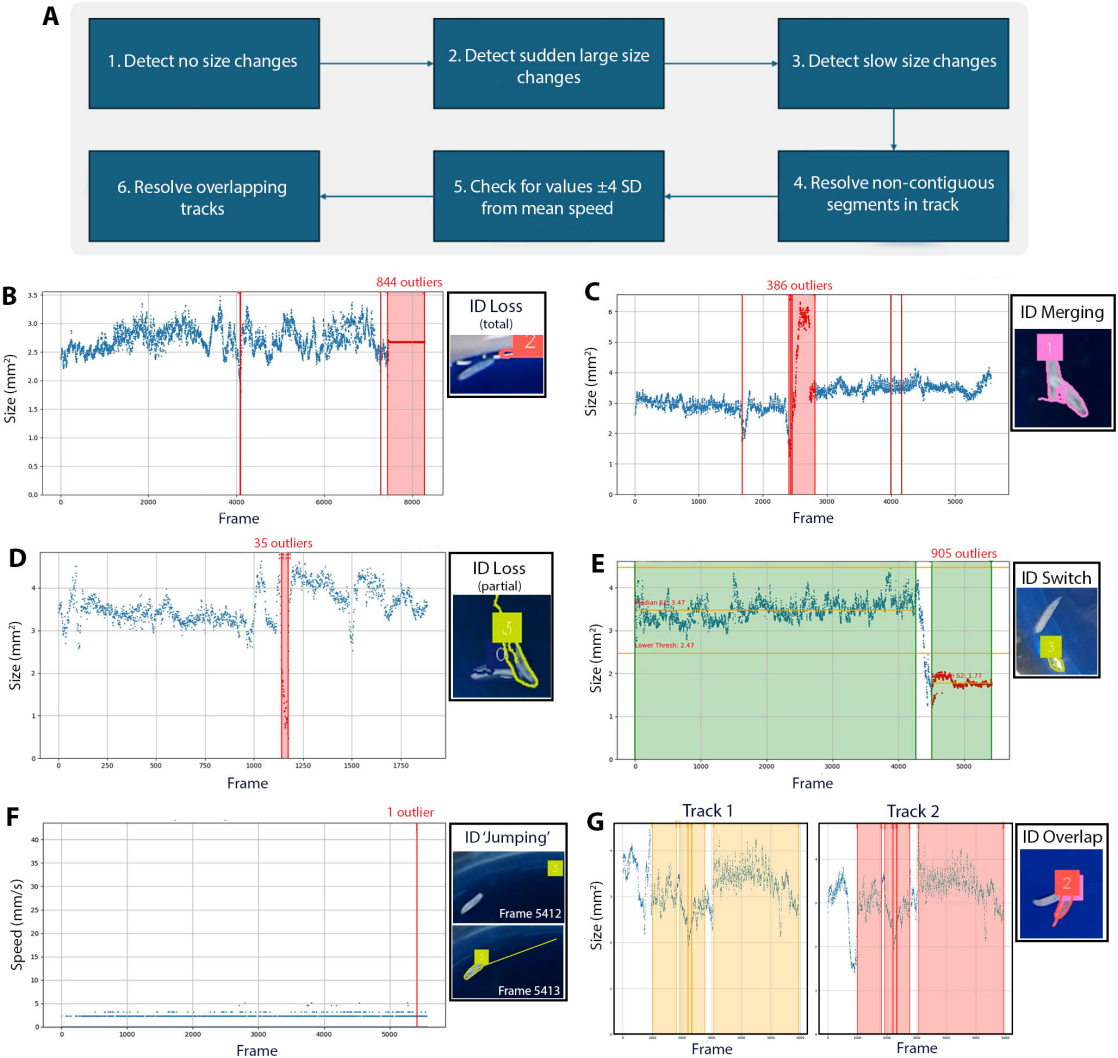
**Post-process error flagging and resolution in Segment Anything Model for Behavioural Analysis (SAMBA) captures tracking errors.** (A) Flowchart of the error detection and resolution process. (B) Continuous frames of no size change for more than 20 frames are flagged. For cases in which the track completely detaches from an animal (3% of cases). (C) Flagged frames that had an absolute size difference greater than 1.5 mm^2^. For cases of sustained animal collision causing merging of IDs (9% of cases). (D) Flagged frames that had a consistent change in size in the same direction relative to the previous frame. For cases in which the tracking polygon shrinks during collision (12% of cases). (E) All the frames within the latter segment are flagged, the median size of the segment did not match the first segment. For cases of ID switching to a new object (5% of cases). (F) Any frames that have a speed value of ±4 s.d. from the mean are flagged. For cases of ID ‘jumping’ between objects. Occurs in conjunction with other error types. (G) Duplicate track detection (1% of cases). Segments of the track are shaded as green (inlier), red (outlier) or orange (duplicate).

To minimise artifacts from segmentation errors, we implemented a robust error identification and handling pipeline for the most common tracking errors (Fig. 3A). As the tracking errors are intrinsic to the SAM2 model, we were limited to addressing them through post-processing. Tracking errors include loss of the animal (the track detaches and remains stationary) and collisions in which two larvae are treated as a single object. SAMBA applies a series of post-processing checks to detect these failure modes (Fig. 3B-G). First, it flags tracks for which object area remains constant for an extended period, indicating that the track has detached from the moving larva and is locked onto the background (3% of cases; Fig. 3B). Second, it detects abrupt increases in object area greater than 1.5 mm^2^, which are characteristic of sustained collisions in which two animals are merged under a single ID (9% of cases; Fig. 3C). Third, it tests for gradual size drift over time. During prolonged contact between animals, an ID can slowly transfer from one individual to another, producing a progressive change in object area (12% of cases; Fig. 3D). To identify sudden ID swaps, SAMBA searches for outlier segments in which median object area deviates from that of the first 200 frames of the track (5% of cases; Fig. 3E). It also captures ‘ID jumping’ events by removing frames with instantaneous speed values more than ±4 s.d. from the track mean (Fig. 3F). Finally, SAMBA detects duplicate tracks and retains the track with the fewest problematic frames (1% of cases; Fig. 3G). All thresholds are user configurable (see Materials and Methods for details).

In a manually curated dataset of 500 larval tracks, 31% contained at least one tracking failure, in line with reported rates from other tracking software (Thane et al., 2023). However, SAMBA correctly detected and removed erroneous data in 97% of these cases. We note that removing frames causes shorter track lengths compared to a track with no erroneous frames, leading to shorter distances. The number of frames removed is provided in the aggregated data summary and visualised in autogenerated scatterplots for each larval track. This allows the user to decide whether to omit tracks from analysis. Typically, we omit any tracks in which more than a third of the frames contain erroneous data.

### Optimisation and validation of SAMBA

Next, we sought to optimise processing time by modifying video frame rate, video resolution and number of objects tracked per video. The current standard video recording framerate on modern devices is ∼30 fps; however, analysing videos at 30 fps in SAMBA increases processing time for no appreciable increase in tracking resolution. Therefore, we included the option in SAMBA to reduce the framerate to a custom value prior to analysis. Videos at 10 fps reduced the processing time per video to ∼5 min, regardless of number of objects or video resolution (Fig. S1A,B). We reran all analyses on mutant lines at 10 fps (unless otherwise stated), and there was no impact on any statistical comparisons or biological conclusions. We also saw that increasing the number of tracked objects increases the number of collision events and, presumably, the number of tracking errors (Fig. S1C). All subsequent analyses were performed with five animals at 10 fps. We used video lengths of 3 min as recordings of between 1 and 4 min are sufficient to capture larval behaviour patterns (Aleman-Meza et al., 2015; Brooks et al., 2016; Risse et al., 2014; Thane et al., 2023).

To evaluate the flexibility of SAMBA, we analysed larval movement on different backgrounds and lighting conditions. We found that a high-contrast (food dye) background was not required for successful tracking and that standard diffuse overhead lighting conditions that avoid bright reflections were sufficient (Fig. S2).

**Fig. 4.**
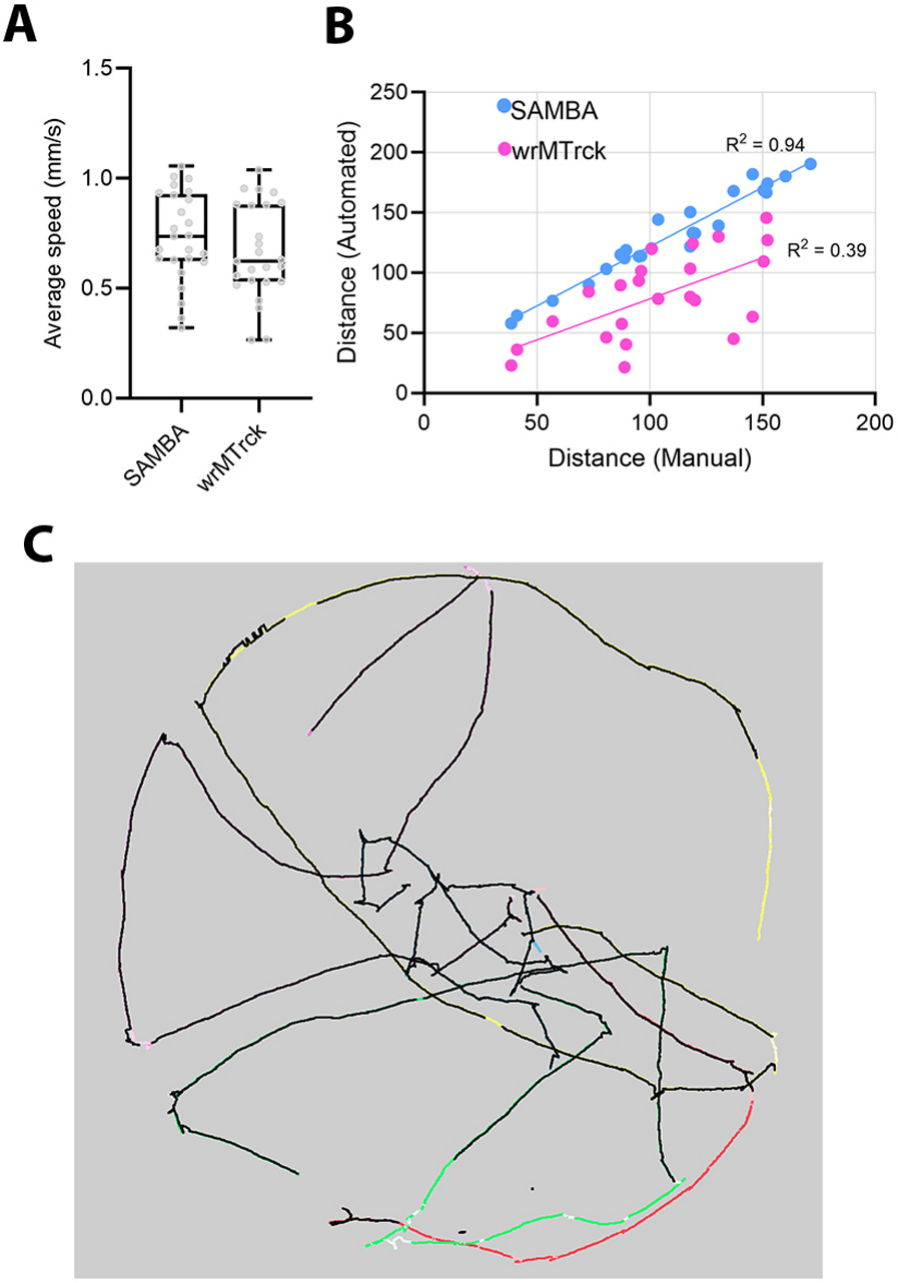
**Tracking accuracy of SAMBA.** (A) Average speed values obtained in SAMBA and wrMTrck. Boxes indicate the 25th to 75th percentiles. Whiskers represent minimum and maximum values. (B) Distance values obtained in SAMBA compared to manual annotation, contrasted with distance values obtained in wrMTrck. Five videos containing five larvae each were used. (C) Overlaid larval movement traces of one video obtained from SAMBA and wrMTrck. SAMBA produced more contiguous tracks (colours) than wrMTrck (black).

To assess the accuracy of SAMBA, we obtained distance measurements from five videos via manual annotation and a functionally similar larva tracker, wrMTrck (Brooks et al., 2016). Although average larval speeds were similar between the trackers (Fig. 4A), distances measured using SAMBA far more closely reproduced values than manual annotation (SAMBA *R*^2^=0.94 versus wrMTrck *R*^2^=0.39; Fig. 4B). However, we observed that SAMBA marginally, but consistently, overestimated distance due to oversampling, an effect that was diminished after reducing the frame rate to 10 fps. Lastly, SAMBA produced more contiguous larval tracks relative to wrMTrck (Fig. 4C).

To further minimise hands-on time, we included a batch processing mode for SAMBA, which allows users to process an entire directory of videos following point prompting in all videos prior to the initiation of tracking. Batch-processing file limits are constrained by the 2-h maximum running time enforced by Google. A total of 15 3-min videos (five animals, 10 fps, 720p) can be analysed in one batch process run. Analysis throughput can be further improved by running multiple instances of SAMBA in parallel, because several Google Colab sessions can be run from the same account provided that the available RAM pool is not exceeded.

### SAMBA for *Drosophila* disease modelling

To evaluate the applications of SAMBA, we used it to analyse three *Drosophila* models of human inherited metabolic diseases with known locomotor deficits. The models represent HIBCH deficiency, MSUD and ISOD (Li et al., 2025; Martelli et al., 2024a; Otto et al., 2018; Tsai et al., 2020). These models were selected because they represent defects in distinct neurometabolic pathways; branched-chain/valine (HIBCH, BCKDHA) and sulfur amino-acid catabolism (ISOD), allowing us to demonstrate SAMBA's applicability across metabolically diverse disease contexts. We selected knockout alleles of the genes encoding each enzyme – *Hibch*, *Bckdha* and *shop*, respectively – resulting in mitochondrial abnormalities and subsequent neurological dysfunction (Claerhout et al., 2017; Taura et al., 2023; Tsai et al., 2020). This, together with our interest in inherited metabolic disorders, made them good candidates for demonstrating the functionality of SAMBA.

**Fig. 5.**
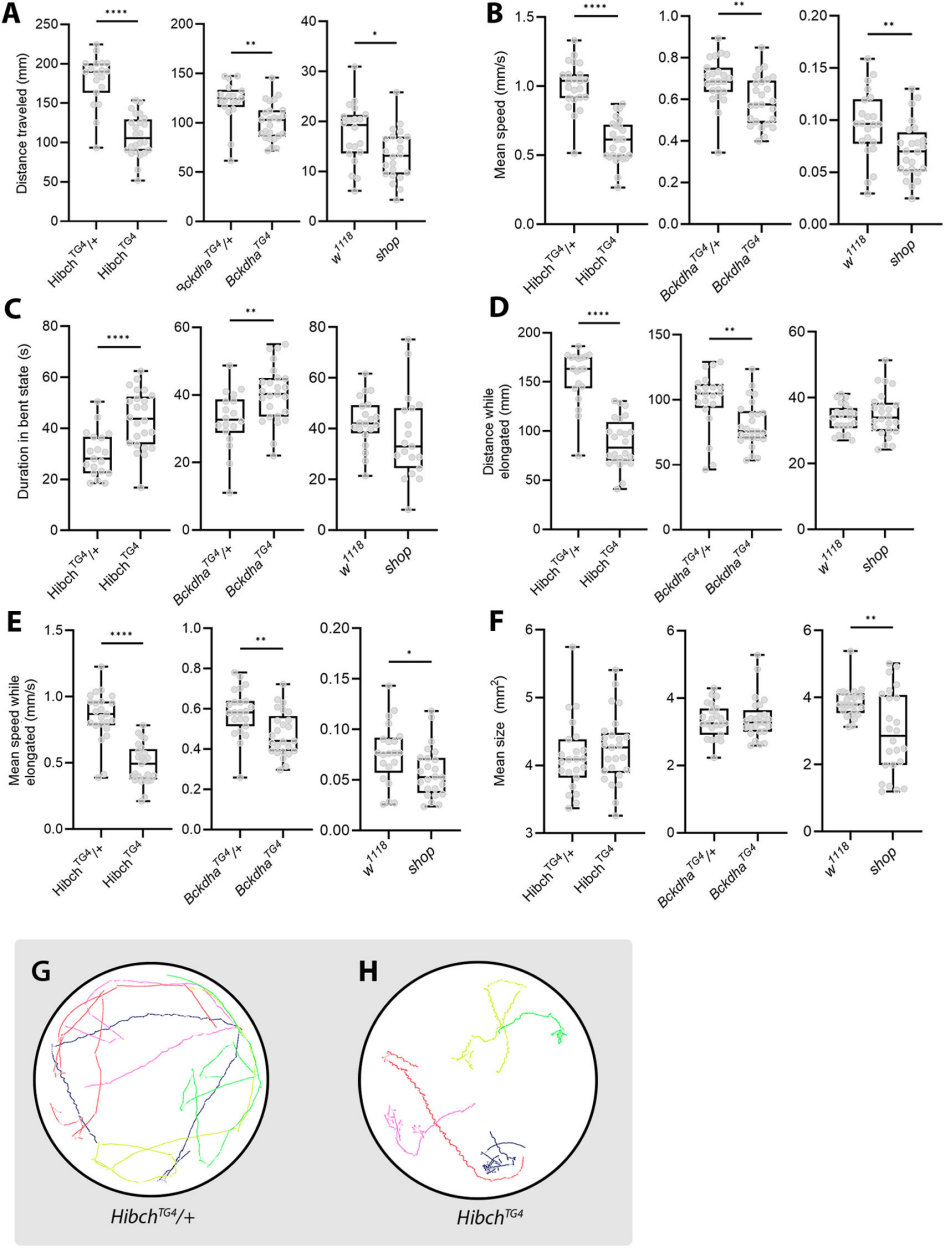
**Locomotor deficits are observed in three *Drosophila* models of neurological disease.** (A,B) Larvae homozygous (*Hibch^TG4^*, *Bckdha^TG4^*) or hemizygous (*shop^C15^*) for mutations in neurological disease gene orthologues travel shorter distance (A) and are slower (B) than heterozygous or wild-type (*w^1118^*) controls. (C) Larvae homozygous for *Hibch^TG4^* and *Bckdha^TG4^* spent more time stationary than heterozygous or wild-type (*w^1118^*) controls, with increased head bending. (D,E) Distance travelled (D) and mean speed (E) adjusted for when a larva is in an elongated state only. (F) Size of larvae. Hemizygous *shop^C15^* larvae were smaller than controls; distance and speed measurements were normalised by larval length. (G,H) Representative traces of larval paths (five larvae per plate); lighter line colour within individual paths indicates that the larva is in a bent state. A total of 25 larvae were recorded per genotype. Boxes indicate the 25th to 75th percentiles. Whiskers represent minimum and maximum values. Unpaired two-tailed Student's *t*-test (****P<0.0001, **P<0.01, *P<0.05).

SAMBA identified significant locomotor impairment in *Hibch*, *Bckdha* and *shop* mutant larvae in overall distances travelled and speed compared to age-matched controls (Fig. 5). SAMBA further identified that *Hibch* and *Bckdha* larvae spent longer in the ‘bent’ state than did controls (Fig. 5C), reflecting short movement paths with frequent turns (Fig. 5G,H), indicative of indecision or impaired exploratory control. After accounting for the time spent in the ‘bent’ state, distance travelled and mean speed remained significantly reduced in the *Hibch*, *Bckdha* and *shop* larvae compared to age-matched controls (Fig. 5D,E). These findings are in line with adult locomotor deficits observed when *Hibch* is knocked down, and larval locomotor deficits seen in *shop* mutants using the wrMTrck method (Li et al., 2025; Martelli et al., 2024a,b). We note that the *shop* distance and speed dataset values were lower than those for *Hibch* and *Bckdha*, likely owing to their rearing on a fully synthetic diet (Martelli et al., 2024b). The *Bckdha* locomotion deficit is consistent with results seen with mutants for *Dbct*, a gene that encodes a subunit in the same enzyme complex as Bckdha (Tsai et al., 2020). These data extend the known phenotypes for *Drosophila* HIBCH and BCKDHA deficiency, highlighting the capacity of SAMBA to detect behaviourally relevant impairments.

### Adaptability of SAMBA to other model systems and organisms

**Fig. 6.**
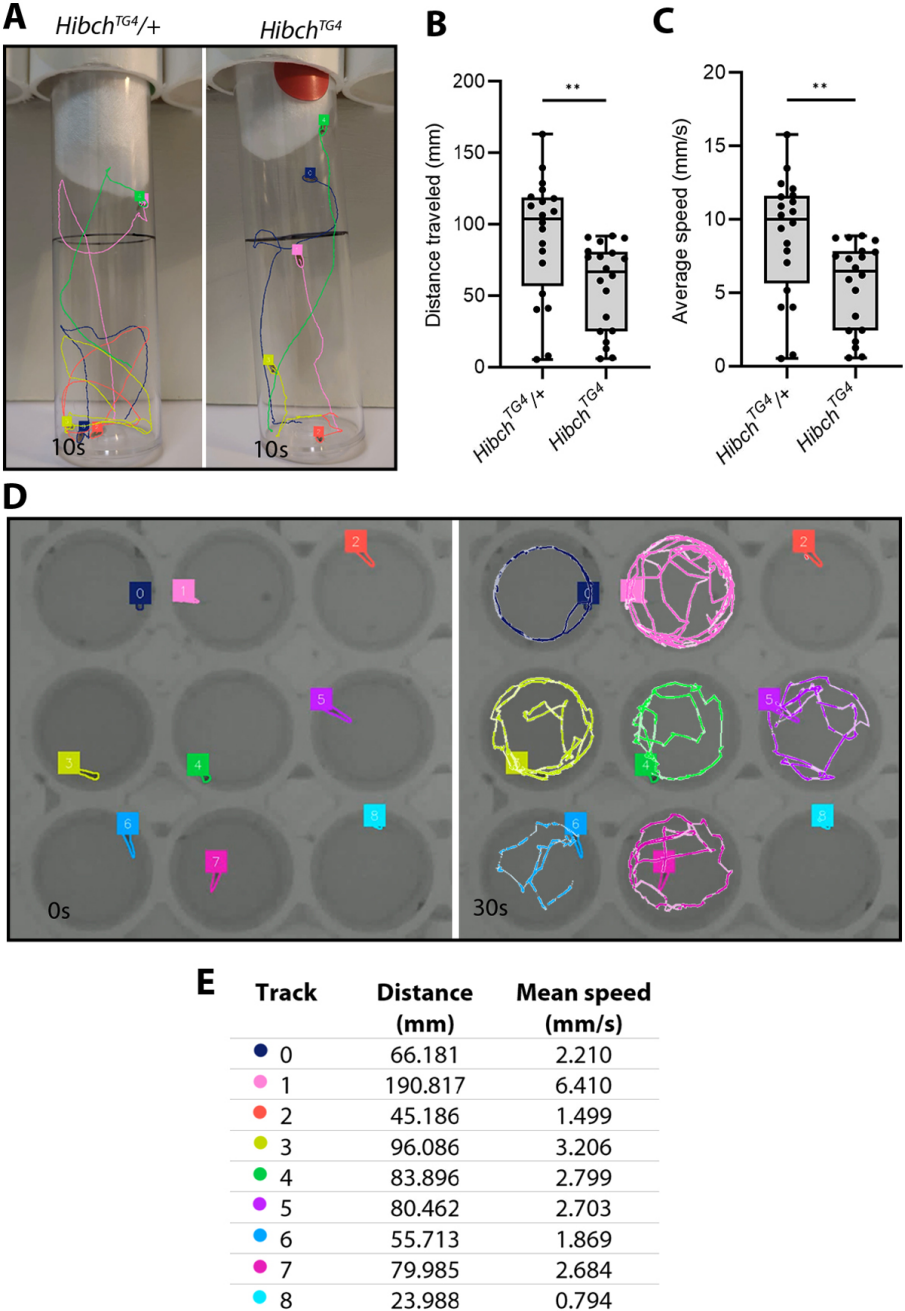
**SAMBA tracking adult *Drosophila* and zebrafish larvae.** (A) Representative adult *Drosophila* negative geotaxis assay. Flies were tapped to the bottom of the vial, and movement was recorded for 10 s then annotated with SAMBA. (B,C) Distance travelled (B) and average speed (C) of 20 flies per genotype. Boxes indicate the 25th to 75th percentiles. Whiskers represent minimum and maximum values. (D) Example of zebrafish larvae movement tracking. Zebrafish were recorded in 48-well plates for 30 s. (E) Movement parameters of tracked zebrafish. Videos were analysed at 30 frames per second. Unpaired two-tailed Student's *t*-test (**P<0.01).

Because SAMBA harnesses SAM2 for object tracking, it has potential for broad applications in behavioural analysis. As a preliminary test of this, we trialled SAMBA for tracking adult *Drosophila* and zebrafish larval movement. Adult *Drosophila* are commonly analysed by a negative geotaxis assay, which is measured by tapping flies to the bottom of a vertical vial and recording the time taken for them to climb a defined distance up the wall, exploiting their innate escape response to gravity (Fergestad et al., 2006). SAMBA could track individual flies in groups of five and calculate their speed and distance travelled, supporting its potential for more accurately quantifying this trait (Fig. 6A-C). Finally, we also applied SAMBA to the analysis of zebrafish larvae [5 days post-fertilisation (dpf)] housed individually in a 48-well plate, a widely used method for phenotyping genetic models, screening neuroactive compounds, or evaluating environmental or metabolic perturbations (Hernández et al., 2024; LaCoursiere et al., 2024; Lara and Vasconcelos, 2021). Here, SAMBA was able to track the larvae and capture speed and distance travelled by these fish (Fig. 6D,E). These preliminary tests illustrate the flexibility of SAMBA for behavioural assays across model organisms.

## DISCUSSION

In this study, we introduce SAMBA, an open-access tool that harnesses the generalist capabilities of SAM2 to detect and track *Drosophila* larvae with no preprocessing and minimal technical setup. We show that SAMBA is ideal for short (< 3 min) behavioural experiments to assess motor pattern defects. Our results demonstrate that SAMBA reliably identifies key features of larval movement, including continuous running, pauses and head-casting behaviours, which are components often associated with decision making or sensory integration. Unlike existing tools, SAMBA does not require thresholding or background subtraction to isolate animals, meaning that videos taken directly from a smartphone, webcam or other low-cost device can be analysed without preprocessing. The tool supports a range of common video file formats (including .MP4, .MOV and .AVI), allowing for integration into diverse experimental setups. SAMBA also features a batch-processing mode, allowing analysis of multiple videos concurrently, with no additional input once configured.

Our analysis of three *Drosophila* models of neurological disease illustrate SAMBA's capacity to detect locomotor deficits. We observed reduced distance travelled, slower average speed and altered exploratory behaviours in mutant larvae, supporting its application for modelling complex neurometabolic conditions. This underscores its utility for detecting not only gross motor defects, but also more nuanced shifts in behavioural state and decision making.

The generalisability of SAM2 opens the possibility of adapting this pipeline to other model organisms (e.g. adult zebrafish, *Caenorhabditis elegans* or even small vertebrates) as shown by our preliminary tests with adult *Drosophila* and zebrafish larvae. SAMBA can be adapted to different contexts using the modifiable parameters embedded in the script (ellipse *R* ratio cut-off, outlier detection). More specific modifications are possible for experienced programmers owing to directly accessible code. This makes SAMBA a versatile platform for applications in genetic screens, nutritional studies, pharmacological testing or environmental stress experiments.

There are several existing larval or small-animal trackers available with widely different requirements and capabilities (Table 2). More feature-rich platforms such as FIMtrack, Multi-Animal Gait and Track (MAGAT) and IMBA tracker have hardware requirements or technical hurdles regarding installation, whereas the more accessible wrMTrck or PEDtracker are limited in scope by their reliance on ImageJ, requiring short videos or very low framerates (three frames per minute in the case of PEDtracker). Furthermore, trackers designed for specific measurements such as the odour attractant response or chemotaxis analysis [MAGAT, Multi-Animal Tracker (MAT), Multi-Worm Tracker] require a MATLAB/LabView licence. In comparison, SAMBA is a simple locomotion deficit detector requiring no installation that can provide fast reliable phenotyping for disease models.

**Table 2.** Overview and comparison of small-animal tracking software

Tracker	Application	Capabilities	Requirements	Reference
SAMBA	*Drosophila* larvae	Positional tracking, bending	Google Colab	This paper
wrMTrck	*Drosophila* larvae	Positional tracking only	ImageJ	Brooks et al., 2016
FIMtrack	*Drosophila* larvae	Object contour, angles, acceleration, bending, stop and go phases, stimulus analysis	Custom-made FIM table	Risse et al., 2014
IMBA	*Drosophila* larvae	Bending/head casting, angular speed, stimulus analysis	Ubuntu v 18.04	Thane et al., 2023
MWT*	*C. elegans*	Bending, shape metrics, chemotaxis, response to tap	LabView, capture card	Swierczek et al., 2011
Ctrax	Adult *Drosophila*	Orientation, walking behaviour patterns	Custom-built arena	Branson et al., 2009
MAGAT^‡^	*Drosophila* larvae	Body alignment, bending, stimulus analysis	MATLAB, custom-built device, bash	Gershow et al., 2012
Maggot Tracker	*Drosophila* larvae	Single-animal positional tracking, bending, contraction rate	Motorised stage	Aleman-Meza et al., 2015
PEDtracker	*Drosophila* larvae	Single-animal staging (3 fpm), size	R, ImageJ	Schumann and Triphan, 2020
Larvaworld	*Drosophila* larvae	Simulate virtual larvae	Python	Sakagiannis et al., 2025
Trex	Sunbleak, zebrafish	Acceleration, angle, visual field, posture	Mid-range Windows PC, Conda	Walter and Couzin, 2021
MAT^§^	*C. elegans*	Chemotaxis, bearing, region of interest	MATLAB, MathWorks camera	Itskovits et al., 2017

fpm, frames per minute; MAGAT, Multi-Animal Gait and Track; MAT, Multi-Animal Tracker; SAMBA, Segment Anything Model for Behavioural Analysis; WMT, Multi-Worm Tracker.

An inherent limitation of SAMBA's reliance on SAM2 is a lack of collision detection and resolution. We instead employed a post-processing approach that likely reduces the amount of usable data compared to trackers that can resolve collisions (IMBA, MAT). We reason that its accuracy and ease of use enables more animals to be assessed, even if a small number are not analysed owing to tracking errors. Another limitation is that SAMBA requires point prompting by the user as opposed to automatic object detection, which adds to user input time. We further recognise that the computational cost associated with running foundation models like SAM2, which require access to an advanced GPU for efficient processing, is also a limitation. Although Google Colab offers a convenient hosted environment, users currently require paid access to more powerful GPU runtimes. The minimum access price as of December 2025 is AUD$15.13, and the cost of analysing one 3-min video within a reasonable timeframe, tracking five objects at 10 fps, is ∼AUD$0.09.

In the future, SAMBA may include more detailed behavioural metrics, such as preference for right versus left bends, speed per cast, total wall-following versus open field exploration and inter-individual distance. Currently, the raw CSV metrics provide detailed frame-by-frame data that could be exported to other analysis programs, while the SAMBA code can be customised to deliver user-specific metrics.

SAMBA may substantially reduce hands-on analysis time, enabling researchers to reallocate effort toward experimental design, data interpretation or follow-up assays. This tool therefore offers an efficient and accessible approach for larval movement analysis, well suited for quick phenotyping with minimal technical setup or expertise. By lowering technical barriers, SAMBA may help broaden the scope of behavioural genetics studies, particularly in models of neurological disease.

## MATERIALS AND METHODS

### *Drosophila* lines and maintenance

The following fly stocks were obtained from the Bloomington (BL) *Drosophila* Stock Center: *y*^1^, *w*^1118^ as the wild-type strain (BL6598); *y*^1^ w*; *Hibch^TG4^*/TM3,Sb^1^Ser^1^ (BL93364); *Bckdha^TG4^*/TM3,Sb^1^Ser^1^ (BL92719); *shop^C15^* (kind gift from Christian Klämbt, Institute for Neuro- and Behavioural Biology, University of Münster, Münster, Germany); FM7a, sChFP (BL35522) and P150/ChFP-TM3, Sb [mCherry fluorescent protein (ChFP) balancer] (BL35524). The mutant alleles were balanced over TM3, ChFP (*Hibch^TG4^* and *Bckdha^TG4^*) or FM7a sChFP (*shop*), and mutant larvae were selected based on the absence of ChFP using a Leica M165FC fluorescent stereo microscope. Flies were maintained on standard sugar-yeast food at 25°C in 12 h:12 h light:dark conditions.

### *Drosophila* larva staging and collection

Embryos were collected from population cages containing 80 females and 20 males and allowed to lay for 8 h on apple juice agar supplemented with yeast paste. Eggs were collected and washed with distilled water, and ∼10 µl of embryos were deposited onto vials containing sugar-yeast media or synthetic media (Martelli et al., 2024b), incubated at 25°C in 12 h:12 h light:dark conditions until larvae reached the third-instar wandering phase. Larvae were removed from the vial by washing it with PBS and gently disturbing the food with a paint brush. Contents were poured into a large Petri dish lined with paper towel; then, using a blunt metal probe, larvae were transferred to a tracking arena.

### Zebrafish husbandry and embryo collection

Wild-type *Danio rerio* (Tübingen strain) were maintained under standard laboratory conditions at La Trobe University, in accordance with institutional animal ethics approvals (AEC16-091, AEC23-011). Adult zebrafish (>3 months of age) were housed in a Tecniplast recirculating aquaculture system and maintained under a 14 h:10 h light:dark cycle. To induce spawning, males and females were separated overnight using a transparent divider. Following a 10-h dark period, lights were turned on to simulate dawn, and the divider was removed to allow mating. Fertilised embryos were collected within 30 min post-fertilisation, rinsed in E3 embryo medium (5 mM NaCl, 0.17 mM KCl, 0.33 mM CaCl_2_, 0.33 mM MgSO_4_) and incubated at 28°C.

### Environment setup and video acquisition

For *Drosophila*, tracking arenas consisted of 60 mm Petri dishes containing apple juice agar dyed dark blue to enhance contrast (see Fig. S2 for Petri dish media that are compatible with SAMBA). Petri dishes were positioned underneath a USB camera (Logitech HD Webcam) held by a retort stand at a height that captured the arena fully in the frame on a dark surface (for setup schematic, see Lin et al., 2024). Videos were recorded under diffuse overhead room lighting, and the setup was positioned such that there were no bright reflections/spots on the agar. These can produce collision-like events when crossed by a larva (Fig. S2E). Larvae were left to acclimate to the arena for 3 min; this also allowed time for larvae to separate if they were transferred all at once. Recording only began when there were no overlapping or touching larvae. If two or more larvae are touching at the start of the video, SAMBA may not differentiate the two objects. Five Petri dishes per genotype were sufficient for this analysis. A ruler was also captured in the frame for calibration. Three-minute videos were recorded. Statistical analyses of behavioural outputs were performed using GraphPad Prism (v10.4.2).

For adult *Drosophila*, five *Hibch* or heterozygous control flies were placed into a vial and dropped down a tube from a 1 m height. Videos were recorded in portrait orientation for 10 s on a Google Pixel 7a phone camera.

For zebrafish, larvae were recorded at 5 dpf in 48-well plates, one larva per well in 1 ml E3 medium. Animals were acclimated for ≥30 min before testing. All experiments were performed in the DanioVision Observation Chamber (Noldus), with environmental temperature held at 28°C and data captured using a GigE digital camera with an infrared lens.

### SAMBA development

SAMBA uses SAM2 to automatically generate a binary segmentation mask for each object in a frame. This mask is used to update the ‘inference state’ of SAM2, which is used for generating predictions in subsequent frames. To extract object measurements, we first generated a polygon surrounding each object (using their binary masks), then computed the geometric centre (centroid) of each polygon using the Python shapely library (Gillies et al., 2025). With the mask, polygon and centroid, we were able to extract per-object measurements, such as the object pixel size (by summing up the number of foreground pixels in the mask), and various movement-based parameters, such as overall distance travelled, speed and orientation (using the object centroid). These outputs were also used to generate different visualisations, such as the visual trace image of the object position across the entire video (using per-frame object centroids) and the video overlay outputs (using per-frame polygons and centroids).

To capture *Drosophila* larval decision making, we used information on larval shape because head-casting behaviour involves body bending. After extracting object contours from the binary mask, we fitted an ellipse [OpenCV fitEllipse() function], which provided the ellipse centre point, major and minor axis lengths, and their orientation angles. The major-to-minor axis ratio (*R*) was used to determine whether the larva was in an ‘elongated’ or ‘bent’ state. For classifying state in each frame, we applied a variable *z*-score threshold to the *R* distribution for each larva across the video. We found that a threshold of 0.8 s.d. was suitable to clearly distinguish the two states. We then integrated these states with movement data, allowing us to define speed, distance and time spent in both ‘elongated’ and ‘bent’ states. Overlaying these on the trace images revealed excellent concordance between running behaviour and the ‘elongated’ state, as well as direction changes (decisions) and the ‘bent’ state.

By default, SAM2 holds the inference state for all video frames in memory, as it uses predictions from previous frames to inform predictions for the next frame. As such, this can lead SAM2 to exceed the available memory on a machine when there are many frames in a video. We observed this with our larval videos, which were ∼3 min long (∼1800 frames at 10 fps). To ensure that SAM2 could process videos of any length, we reduced its memory usage by retaining only the most recent 1024 frames in the inference state (∼1 min 42 s at 10 fps). We observed that this provided a good trade-off between reducing memory requirements without causing noticeable deterioration in tracking outputs.

The error detection and handling pipeline will first identify whether there is no size change for more than 20 frames. Segments of the track are created which are regions in which a contiguous sequence of frames is considered an inlier or outlier. ‘Infilling’ is then performed in which inlier frames are flagged as outliers if they fall within 20 frames of two neighbouring outlier segments. Next, frames are removed if their absolute size difference is greater than 1.5 mm^2^. This occurs in cases of sustained animal collision leading to merging of object IDs. To detect slow size changes, a median filter is applied to the size data (kernel size, 201) to highlight areas of continual increase or decrease. The difference of the signal is also computed and smoothed (kernel size, 11). SAMBA then checks an 80-frame window in the difference signal for whether 75% of the frames change in size in the same direction relative to the previous frame. A segment is kept if the smoothed size value at the start and end of the segment differ by >1 to ensure that there is a difference in statistics before and after the drift. To detect ID switching, the first segment in the track is set as the baseline median if there are at least 200 frames in that segment. Otherwise, the next segment with enough frames is chosen. The medians of all other segments are compared to the baseline median. If they differ by >1, the entire segment is flagged as an outlier. For all remaining inlier frames, frames are flagged as outliers if they are more than ±4 size s.d. from the mean, or more than ±5 speed s.d. from the mean. To detect duplicate tracks, pairwise comparisons are made between the centroids of two objects and if they are identical for more than 100 frames, the track with the most outlier frames is removed. Infilling is done around these regions by 20 frames, as the centroid does not always identically match across objects. If both objects have the same number of outliers, then the track is assigned to the first object.

### Running SAMBA

Instructions are included within the SAMBA Colab notebook. Running SAMBA in Google Colab requires the use of a Google account, as outputs are stored in a Google Drive folder. In SAMBA, the required libraries and helper functions are initialised in Google Colab by running step 1 (setup environment), followed by the creation of working directories in Google Drive by running step 2 (setup working directory), which allows the notebook to read and write files to a personal Google Drive account. To determine the scale factor (mm pixel^−1^), step 3 (calibration) is run, which prompts user selection of two points of known distance in the frame of a video. Alternatively, a predetermined scale factor can be used. The ‘Analysis Option A – Single video’ or ‘Analysis Option B – Batch processing’ sections are used to select a single video or a directory containing several videos, respectively. In batch processing, each video is sequentially previewed and visible larvae on frame 0 (by default) are selected by clicking. Larval tracking is then completed automatically by SAMBA, and the outputs are generated at the specified directories. With minor changes and sufficient processing power, SAMBA could also be run locally.

## Supplementary Material

10.1242/dmm.052631_sup1 Supplementary information
