# Supplementary material for: A Segment Anything Model-based tool for semi-automated behavioural analysis of Drosophila and other model organisms
Source: Dis Model Mech. 2026 Feb 24;19(2):dmm052631. doi: 10.1242/dmm.052631 (PMC12964350; doi:10.1242/dmm.052631)
Supplement: Supplementary information [file dmm-19-052631-s1.pdf]

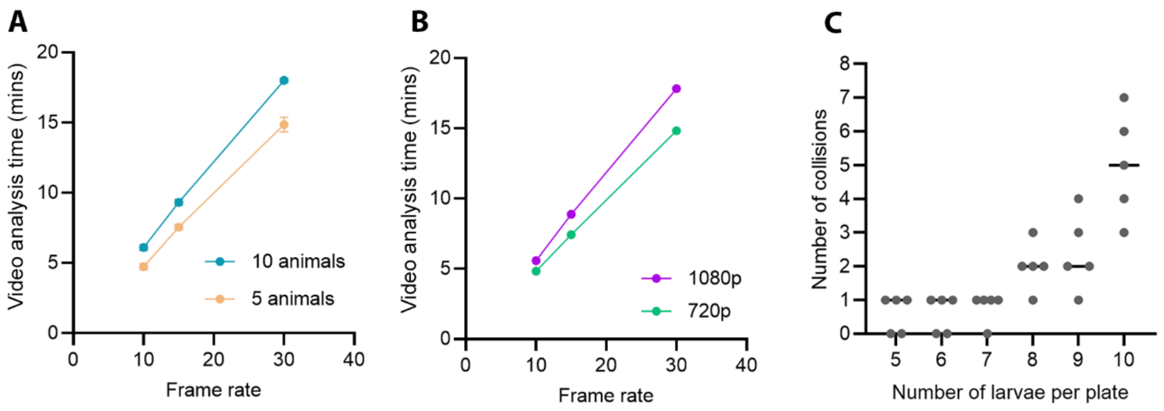

Fig. S1. Optimisation of SAMBA performance for larval locomotor analysis. (**A**) Effect of number of objects tracked and video frame rate on processing time video resolution was 720p. (**B**) Effect of video resolution and frame rate on processing time. Five animals were tracked. GPU: A100. (**C**) Number of larva-larva collisions counted manually during five 3-minute videos per condition.

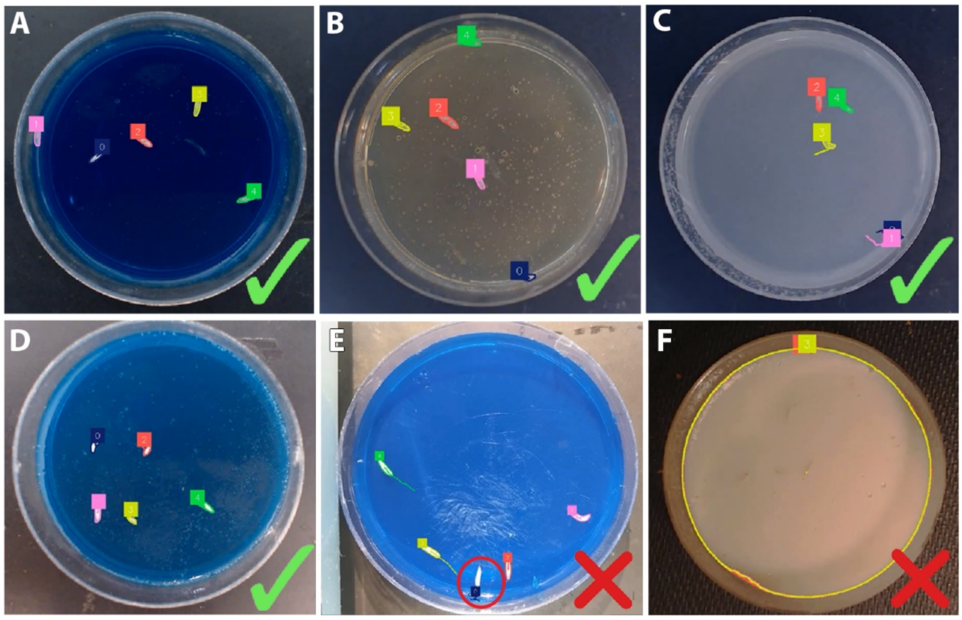

Fig. S2. Acceptable backgrounds and media for use in SAMBA. (**A**) Apple juice agar with dark contrast dye. (**B**) Apple juice agar. (**C**) Plain agar. (**D**) Apple juice agar with contrast dye and surface imperfections. (**E**) Plain agar with contrast dye and bright light reflections. Bright spots can cause tracking errors (circled). (**F**) Sugar-yeast media is not recommended.
